# The Efficacy of Platelet-Rich Plasma in the Repair of Tympanic Membrane Perforation

**DOI:** 10.1055/s-0044-1791646

**Published:** 2025-01-27

**Authors:** Kudure Basavaraj Prashanth, Haraganahalli Anandappa Manjunatha, Prathvi P. Nayak

**Affiliations:** 1 Department of Ear, Nose and Throat, JJM Medical College, Davangere, Karnataka, India

**Keywords:** platelet-rich plasma, myringoplasty, tympanic membrane perforation, ear, otology

## Abstract

**Introduction** Platelet-rich plasma (PRP) contains high platelet concentration and growth factors that help in rapid wound healing, hemostasis, and decreased scarring. It has been used in various conditions to aid in healing, but its use in ear, nose, and throat (ENT) is not yet common.

**Objective** To compare the outcome of using PRP with myringoplasty with that of myringoplasty alone in the repair of tympanic membrane perforations.

**Methods** Sixty-eight patients in the 16-to-50 years age group with tympanic membrane perforation of 3 months duration, with dry ear for 6 weeks, and mild to-moderate conductive hearing loss were selected and divided by simple randomization into 2 equal groups. A thorough preoperative evaluation was done. In group A, patients underwent myringoplasty, and in group B, patients underwent myringoplasty with PRP. Patients were followed up for 3 months postsurgery.

**Results** At 6 weeks, 67.6 and 94.1% had graft uptake in groups A and B, respectively ( *p* -value 0.011). At 3 months, 85.3 and 97.1% had graft uptake in groups A and B, respectively ( *p* -value 0.197). The mean pure tone audiometry difference in group A was 8.35 ± 2.05, and 11.00 ± 2.28 in group B ( *p* < 0.001). The mean air-bone gap difference for group A was 8.29 ± 2.14, and 10.76 ± 2.36 for group B ( *p* < 0.001). Graft uptake rate at 6 weeks, pure tone audiometry, and air-bone gap difference were statistically significant.

**Conclusion** The present study results showed that the use of PRP during myringoplasty aids healing of the tympanic membrane with better hearing improvement in the postoperative period when compared to myringoplasty alone.

## Introduction

Tympanic membrane (TM) perforation can occur due to trauma, eustachian tube dysfunction, or infections, such as acute otitis media (AOM), fungal infections of the ear canal, and chronic suppurative otitis media (CSOM). Tympanic membrane perforation can be acute or chronic depending on the duration. If the perforation is present for a duration of more than 3 months, it is chronic. 1 2 One of the most important causes for chronic TM perforation is CSOM, which leads to significant hearing loss. India belongs to the group of countries with the highest prevalence of CSOM, with 4% and more, representing a public health problem that necessitates urgent attention. 3

Acute and traumatic perforations usually heal spontaneously. 4 5 Chronic tympanic perforation can lead to hearing loss, recurrent ear discharge, and formation of cholesteatoma, and it can affect the quality of life. 1 6 This necessitates the repair of the TM to prevent further infection of exposed middle ear mucosa and destruction of middle ear structures, which could lead to progression of hearing loss. 5 Various techniques have been used in the attempt of repairing TM perforations. Myringoplasty is the conventional method of treatment, in which only the TM is surgically repaired. But the terms myringoplasty and tympanoplasty type 1 have been used interchangeably. Various materials like vein grafts, fat grafts, split and full thickness skin grafts, perichondrium, periosteum, fascia, cartilage graft, and others, have been used in the past to repair TM perforations. Out of these, fascial graft has been used widely because of its internal structure, and it is also easily available for harvesting. 7 Minimally invasive methods, such as the use of chemical cautery, gelfoam, and cigarette papers, have also been attempted. 1 5

The surgical success rate of myringoplasty may range from 64 to 96%, 7 and in a study on conventional underlay myringoplasty done by Mururgendrappa et al. it was found to be 76%. 8 Surgical success is dependent on the optimal contact between the newly placed graft and the remaining TM, and failure can occur due to medialization or lateralization of graft. Any substance that helps in rapid healing will, in turn, help in reducing the chances of graft migration leading to increase in the graft uptake rate. 8 9 10 Fibroblast growth factor and hyaluronic acid have been used in otology to promote healing of TM perforations. 11 Platelet-rich plasma (PRP) is one such product, which has also been known to promote healing and recently has been used in the treatment of TM perforations.

Platelet-rich plasma is a small quantity of autologous blood containing a platelet concentration which is above baseline and contains various growth factors. Some of these growth factors are platelet-derived growth factor, vascular endothelial growth factor (VEGF), insulin-like growth factor (IGF), platelet-derived angiogenesis factors, and transforming growth factor (TGF). These are known to help in hastening the process of wound healing, hemostasis, and in decreasing the amount of scar tissue formed. 9 12 Platelet-rich plasma is already being used as a treatment modality in various other fields of medicine, such as cardiothoracic surgery, orthopedics, neurosurgery, and plastic surgery, among others. 12 It has been successfully used in various conditions, but its application in ENT is not yet well studied.

Myringoplasty is still one of the most used methods to treat chronic TM perforations. But there is a chance of surgical failure with occurrence of reperforation and postoperative otorrhea. Using PRP along with myringoplasty can help in improving the success rates due to its properties of healing and decreased scarring. Hence, the primary aim of the current study is to evaluate and assess the efficiency of using PRP during myringoplasty regarding graft uptake, hearing improvement and postoperative complications.

## Methods

This is a prospective comparative study with a sample size of 68, including patients within the age group of 16 to 50 years. Patients who attended our hospitals with TM perforation, who met all inclusion and exclusion criteria specified, and gave consent to participate in this study were selected. A simple randomization method was used to divide the patients into 2 groups of 34 each. Group A underwent myringoplasty alone and group B underwent myringoplasty with the use of PRP. Written informed consent was obtained from all patients, who also underwent a detailed assessment including thorough history taking and general and systemic physical examination. Additionally, the patients underwent complete ENT examination, which included tuning fork tests and pure tone audiometry (PTA).

Statistical analysis was performed using the IBM SPSS Statistics for Windows, Version 25.0. (IBM Corp., Armonk, NY, USA). Continuous measurements results were depicted as mean ± SD, and categorical as frequency (percentage). Normality of the data was checked using the Shapiro-Wilk test or Kolmogorov-Smirnov test. The Chi-squared or Fischer exact test and Mann-Whitney U test were used to check the difference between the groups. Pre and postvalues within a group were checked using the Wilcoxon Signed-Rank test. The level of significance was 5%.

### Inclusion Criteria

Patients of both sex in the 16-to-50 years age group, with history of TM perforation for no less than 3 months were selected. Criteria of dry ear for at least 6 weeks and having mild-to-moderate conductive hearing loss was also included.

### Exclusion Criteria

Any patients with comorbid conditions, active ear discharge, granulation tissue, atticoantral disease, marginal perforation, acute traumatic perforation, or with mixed hearing loss were excluded.

### Platelet-Rich Plasma Preparation

The PRP preparation was done according to the method explained by Dhurath et al., 13 and a literature based double spin method as explained by Mazzacco et al. 14 A total of 10cc of patient's blood was collected into a tube with acid citrate dextrose. The blood was then centrifuged with a soft spin (1,500 rpm for 5 minutes), separating it into three different layers. The topmost layer was the acellular platelet-poor plasma, the intermediate buffy coat layer contained PRP, and the bottom most layer contained the red blood cells. The top and intermediate buffy coat layers were collected using a syringe and transferred to another tube without any anticoagulant for another round of centrifugation with a hard spin (6,300 rpm for 20 minutes). The topmost acellular layer (platelet-poor plasma) was collected with a syringe and discarded, and the PRP that remained at the bottom of the tube was allowed to settle. The PRP was allowed to stay in the tube, which was used for further treatment in group B.

### Myringoplasty

Myringoplasty was conducted on all patients with temporal fascia graft in a conventional underlay technique. For group A, gelfoam soaked in antibiotic solution was placed laterally to the newly placed graft. For group B, after placing the graft, PRP was applied onto the graft using a tuberculin syringe. Gelfoam soaked in PRP was placed lateral to the graft, circumferentially. Antibiotic-soaked gelfoam was placed laterally to the PRP-soaked gelfoam.

Postoperative follow-up of the patient was done on the 1 ^st^ , 3 ^rd^ , and 6 ^th^ weeks as well as on the 3 ^rd^ month after surgery. In each visit, thorough history was taken, and examination was done to assess the graft uptake and healing rate. On the 3 ^rd^ month follow-up, PTA was performed to check for hearing loss, and air-bone gap was measured and compared with the preoperative results.

### Ethical Standards

The present study was approved by the institutional ethics committee (JJMMC/IEC-Sy-91-2019).

## Results

The current study was conducted with an aim to evaluate the efficacy of PRP in the repair of TM perforations following tympanoplasty regarding graft uptake, hearing improvement, and postoperative complications.

The study sample consisted of 68 patients who were divided into 2 groups, A and B, by simple randomization technique. Each group contained 34 patients of either sex. Group A underwent myringoplasty alone, and group B underwent myringoplasty with the use of PRP. We primarily observed and analyzed the sex distribution, preoperative presenting complaints, size of perforation, graft uptake, pre and postoperative PTA values, and occurrence of complications.

Homogeneous gender distribution was found in both groups, with 17 females and 17 males in both groups A and B ( *p* -value 0.99). The age group ranged from 16 to 50 years, with a mean age of 35. In group A, 16 patients had left ear, and 18 had right ear involvement. In group B, 17 patients had left ear, and 17 had right ear involvement ( *p* -value 0.808). When comparing the presenting complaints, 25 patients (73.5%) had ear discharge, and 29 (85.3%) had hearing loss in group A. In group B, 23 patients (67.7%) presented with ear discharge and 20 (58.8%) presented with hearing loss. In group A, 3 patients had small, 19 had medium, and 12 had large perforation. In group B, 10 had small, 16 had medium, and 8 had larger perforation ( *p* -value 0.90) ( Table 1 ).

**Table 1:** Description of the clinical presentation of the patients (n = 68)

		Group A N (%)	Group B N (%)	*P* -value
**Ear involvement**	Left	16 (47.1)	17 (50)	0.808
	Right	18 (52.9)	17 (50)	
**Ear discharge**	Absent	09 (26.5)	11 (32.3)	0.595
	Present	25 (73.5)	23 (67.7)	
**Hearing loss**	Absent	05 (14.7)	14 (41.2)	0.015*
	Present	29 (85.3)	20 (58.8)	
**Size of perforation**	Small	03 (8.8)	10 (29.4)	0.090
	Medium	19 (55.9)	16 (47.1)	
	Large	12 (35.3)	08 (23.5)	

Note: *Statistically significant difference is observed between the groups for hearing loss as more cases were present in group A.

Group A underwent myringoplasty and group B underwent myringoplasty with PRP. Patients were then followed up. A comparison of postoperative otorrhea incidence in groups A and B showed that the patients in group A who underwent only myringoplasty had more incidences thank those in group B, who underwent myringoplasty with PRP. In group A, the incidence of otorrhea was at 3 weeks (11.8%), at 6 weeks (23.5%), and 3 months (2.9%). In group B, the incidence was 2.9%, 11.8%, and 2.8% at 3 weeks, 6 weeks, and 3 months, respectively. However, these results were not statistically significant ( Table 2 ).

**Table 2:** Number of patients developing postop ear discharge (n = 68)

Discharge from Ear	Group A N (%)	Group B N (%)	*P* -value
**3 weeks**			
Absent	30 (88.20)	33 (97.1)	0.356
Present	04 (11.8)	01 (2.9)	
**6 weeks**			
Absent	26 (76.5)	30 (88.2)	0.340
Present	08 (23.5)	04 (11.8)	
**3 months**			
Absent	33 (97.1)	33 (97.1)	0.99
Present	01 (2.9)	01 (2.9)	

At 6 weeks of postoperative follow-up, a significant difference in the graft uptake was noticed between both groups. In group A, only 23 patients (67.6%) had graft uptake whereas in group B, 32 patients (94.1%) had graft uptake. At the 3-month follow-up, 29 patients (85.3%) had graft uptake in group A, and 33 patients (97.1%) in group B. Even though the values between both groups showed difference, the values were not found to be statistically significant. This shows that PRP aids in rapid healing and, in turn, in better graft uptake ( Table 3 ).

**Table 3:** Graft Uptake rate in both groups (n = 68)

Graft Uptake	Group A N (%)	Group B N (%)	*P* -value
**6 weeks**			
Not Taken	11 (32.4)	02 (5.9)	0.011*
Taken	23 (67.6)	32 (94.1)	
**3 months**			
Not Taken	05 (14.7)	01 (2.9)	0.197
Taken	29 (85.3)	33 (97.1)	

Note: *Statistically significant difference is observed between the groups for graft uptake at 6 weeks

At the 3-month follow-up, PTA was done for all patients, and it was compared with the preoperative PTA values. Hearing improvement was then assessed. Statistically significant difference in hearing improvement was noted between both groups, with group A showing 8.35 ± 2.05 and group B showing a PTA difference of 11.00 ± 2.28. This suggests that PRP helps in healing of the TM and improves hearing.

**Graph 1:**
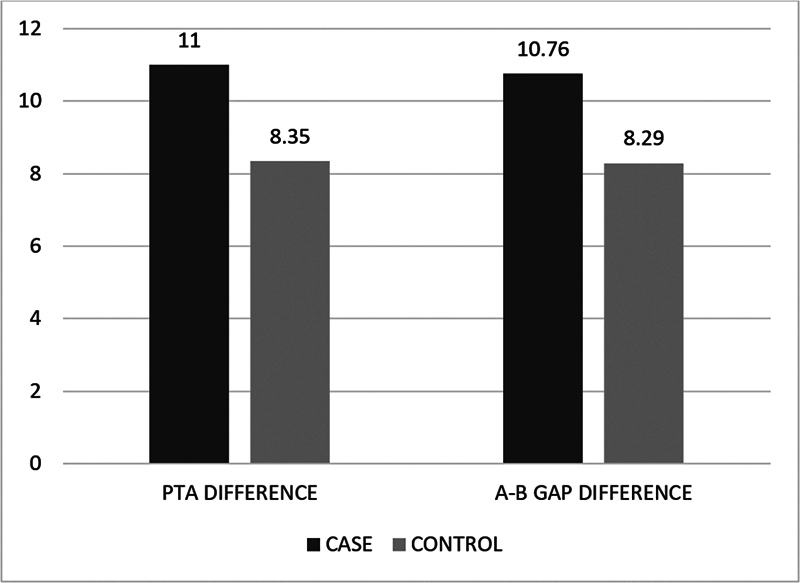


The A-B gap difference was calculated for all patients after comparing their pre and postoperative A-B gap values. It was noted that there was a significant improvement in the A-B gap between both groups. In group B, the difference was found to be 10.76 ± 2.36, which was higher than in group A (8.29 ± 2.14). This shows that there was better hearing improvement in patients who underwent myringoplasty with PRP ( Table 4 ) ( Graph 1 ).

**Table 4:** Comparison of pre and postoperative PTA and A-B gap between case and control group (n = 68)

		Group A (Mean ± SD)	Group B (Mean ± SD)	*P* -value
**PTA**	Preop PTA	40.82 ± 6.77	37.76 ± 7.36	0.075
	Postop PTA	32.47 ± 6.73	26.76 ± 7.45	0.002*
	PTA difference	8.35 ± 2.05	11.00 ± 2.28	0.001*
**A-B gap**	Preop A-B gap	29.03 ± 6.38	26.03 ± 6.26	0.043
	Postop A-B gap	20.74 ± 6.37	15.26 ± 6.34	0.001*
	A-B gap difference	8.29 ± 2.14	10.76 ± 2.36	0.001*

Note: *Significant results

## Discussion

Chronic TM perforation can cause damage to the middle ear leading to repeated episodes of ear discharge and hearing loss. Conventionally, myringoplasty is the surgery performed to close the perforation and, thus, prevent complications. 14 Surgical failure can occur due to displacement of the graft and lead to persistent perforation, ear discharge, and hearing loss. Any substance that aids in rapid healing can help reduce the surgical failure rate. 9 Therefore, there has been a search for new methods which overcome these drawbacks. Platelet-rich plasma is such one adjunct. It is a product derived from autologous blood through the process of centrifugation; it contains high platelet concentration as well as many growth factors, some of which include platelet-derived growth factor, IGF, FGF, and TGF, among others. 6 8 As PRP aids in wound healing and has immunological property that help in rapid wound healing with reduced scarring and infections, combining it with myringoplasty provides a better surgical success rate.

In a double blinded study by Saeedi et al. in patients with chronic TM rupture, the results of type 1 tympanoplasty with PRP-enriched gelfoam and surgery with gelfoam alone were compared. At the 3-month follow-up, complete healing of the TM was seen in 66.67% in the group that underwent tympanoplasty with PRP-enriched gelfoam, which was statistically significant. No patients in the intervention group developed ear discharge in the follow-up period, but 3 patients in the control group developed ear discharge. 1

In a study done by Yadav et al., patients with chronic otitis media were separated into 2 groups, with group 1 patients undergoing myringoplasty with PRP and group 2 patients undergoing myringoplasty without PRP. Group 1 showed a graft uptake rate of 95% whereas group 2 showed 85%, which was statistically significant. In this study, the mean gain in hearing threshold for group 1 was 18.62 dB, for group 2 it was 13.15 dB, and the A-B gap difference in group 1 was 18.62 dB, and for group 2 it was 13.15 dB, which was also statistically significant. 9

A randomized control trial was performed by El-Anwar et al. on 64 patients with TM perforations. The success rate for patients who underwent tympanoplasty with PRP was 100%, which was compared to that of patients who underwent only tympanoplasty, who had a success rate of 81.25%. In this study, no postoperative complications were seen, but 4 patients in the control group had otorrhea that required medical treatment. 14

The results from the studies mentioned earlier can be compared to those of ours. In the present study, 4 patients in the myringoplasty group had otorrhea, and only 1 patient in myringoplasty with PRP group had otorrhea at 3 weeks of follow-up. A total of 97.1% patients who underwent tympanoplasty with PRP and 85.3% patients who underwent only tympanoplasty had complete healing. We found that the mean PTA difference for the case group was 11 ± 2.28 and 8.35 ± 2.05 for the control group, and the air-bone gap difference for the case group was 10.76 ± 2.36 and 8.29 ± 2.14 for the control group.

Better graft uptake rate with the use of PRP with myringoplasty can be explained by the integral property of PRP to aid in healing as well as its adherence, which leads to decrease in graft displacement. The decrease in the postoperative infection rate can be explained by the immunological property of PRP. However, the mechanism for significant improvement in hearing with the use PRP is not currently known. Further research into the hearing improvement due to PRP is needed, preferably with a larger sample size and longer study follow-up period to ascertain the results found in the present study.

## Clinical Significance

Our study showed that using PRP during tympanoplasty increased the complete healing rate, hearing improvement, and decreased postoperative otorrhea. Our study, along with the previous studies mentioned, emphasizes the benefits of using PRP in the TM perforation repair. As this study was conducted during the period of coronavirus disease 2019 (COVID-19), the longer term of follow-up was not possible, and even the sample size of the study was small. Studies with a larger study group, preferably with multiple centers and with longer follow-up term would help in providing more conclusive evidence on the efficacy of PRP in TM repair.

## Conclusion

Platelet-rich plasma contains growth factors which help in healing. In our prospective randomized study, we found that the use of PRP along with myringoplasty for the repair of chronic TM perforation increases the healing rate as well as hearing improvement, and it decreases the incidence of postoperative complications. As PRP is extracted from the patient's own blood, there is no risk of any reactions or infections. And it is also an easy and cheap method which can be used to ensure the success of the surgery.

## References

[1] Saeedi M Ajalloueian M Zare E The effect of PRP-enriched gelfoam on chronic tympanic membrane perforation: a double-blind randomized control trial Int Tinnitus J 2017 21 02 108 111 10.5935/0946-5448.20170021 29336128

[2] Gupta S Harshvardhan R Samdani S To Study the Association of the Size and Site of Tympanic Membrane Perforation with the Degree of Hearing Loss Indian J Otolaryngol Head Neck Surg 2019 71(2, Suppl 2)1047 1052 10.1007/s12070-017-1102-9 31750125 PMC6841911

[3] WHO/CIBA Foundation workshop (1996: London, United Kingdom), World Health Organization.Programme for the Prevention of Deafness and Hearing impairment & CIBA Foundation 1998). Prevention of hearing impairment from chronic otitis media. Report of a WHO/CIBA foundation workshop, World Health Organization, London, U.K., pp 19–21.https://apps.who.int/iris/handle/10665/63870

[4] Karataylı Özgürsoy S Tunçkaşık M E Tunçkaşık F Akıncıoğlu E Doğan H Kocatürk S Platelet-Rich Plasma Application for Acute Tympanic Membrane Perforations J Int Adv Otol 2017 13 02 195 199 10.5152/iao.2016.2533 28084996

[5] Santhi T Rajan K V A study of closure of tympanic membrane perforations by chemical cauterisation Indian J Otolaryngol Head Neck Surg 2012 64 04 389 392 10.1007/s12070-011-0425-1 24294587 PMC3477427

[6] El-Anwar M W Elnashar I Foad Y A Platelet-rich plasma myringoplasty: A new office procedure for the repair of small tympanic membrane perforations Ear Nose Throat J 2017 96 08 312 326 10.1177/014556131709600818 28846786

[7] Sarkar S A review on the history of tympanoplasty Indian J Otolaryngol Head Neck Surg 2013 65 03 455 460 10.1007/s12070-012-0534-5 24427697 PMC3889360

[8] Murugendrappa M A Siddappa P N Shambulingegowda A Basavaraj G P Comparative study of two different myringoplasty techniques in mucosal type of chronic otitis media J Clin Diagn Res 2016 10 02 MC01 MC03 10.7860/JCDR/2016/16843.7194 PMC4800556 27042491

[9] Yadav S PS Malik J S Malik P Sehgal P K Gulia J S Ranga R K Studying the result of underlay myringoplasty using platelet-rich plasma J Laryngol Rhinol Otol 2018 132 11 990 994 10.1017/S0022215118001846 30370872

[10] Navarrete Álvaro M L Ortiz N Rodriguez L Pilot study on the efficiency of the biostimulation with autologous plasma rich in platelet growth factors in otorhinolaryngology: otologic surgery (tympanoplasty type I) ISRN Surg 2011 2011 451020 10.5402/2011/451020 22084757 PMC3199916

[11] Ribeiro L Castro E Ferreira M The concepts and applications of tissue engineering in otorhinolaryngology Acta Otorrinolaringol Esp 2015 66 01 43 48 10.1016/j.otorri.2014.03.007 25440936

[12] Stavrakas M Karkos P D Markou K Grigoriadis N Platelet-rich plasma in otolaryngology J Laryngol Rhinol Otol 2016 130 12 1098 1102 10.1017/S0022215116009403 27938467

[13] Dhurat R Sukesh M Principles and Methods of Preparation of Platelet-Rich Plasma: A Review and Author's Perspective J Cutan Aesthet Surg 2014 7 04 189 197 25722595 10.4103/0974-2077.150734 PMC4338460

[14] El-Anwar M W El-Ahl M A Zidan A A Yacoup M A-R Topical use of autologous platelet rich plasma in myringoplasty Auris Nasus Larynx 2015 42 05 365 368 10.1016/j.anl.2015.02.016 25794691

